# Cyclosporine A eyedrops with self-nanoemulsifying drug delivery systems have improved physicochemical properties and efficacy against dry eye disease in a murine dry eye model

**DOI:** 10.1371/journal.pone.0224805

**Published:** 2019-11-18

**Authors:** Seung Pil Bang, Chang Yeor Yeon, Nirpesh Adhikari, Sanjiv Neupane, Harim Kim, Dong Cheol Lee, Myeong Jin Son, Hyun Gyo Lee, Jae-Young Kim, Jong Hwa Jun

**Affiliations:** 1 Department of Ophthalmology, Keimyung University School of Medicine, Dongsan Medical Centre, Daegu, Republic of Korea; 2 Department of Biomedical Engineering, University of Rochester, Rochester, New York, United States of America; 3 Department of Biochemistry, School of Dentistry, IHBR, Kyungpook National University, Daegu, Republic of Korea; University of Rochester School of Medicine and Dentistry, UNITED STATES

## Abstract

**Purpose:**

We aimed to compare the physicochemical properties and *in vivo* efficacy of commercially available nanoemulsion cyclosporine A (CsA) eyedrops in benzalkonium chloride (BAC)-induced dry eye disease (DED).

**Methods:**

Particle size analysis was performed on conventional 0.05% CsA (Restasis, C-CsA) and two new types of 0.05% CsA eyedrops based on a self-nanoemulsifying drug delivery system (SNEDDS, SNEDDS-N and -T). Turbidometry, pH measurements and instability indices of each CsA solution were measured. DED was induced with BAC, and animals were treated with vehicle or CsA preparations. Tear volume and fluorescein staining scores were evaluated on days 7 and 14. Eyes were enucleated and subjected to IHC, TUNEL staining, periodic acid-Schiff (PAS) staining, real-time PCR and western blotting.

**Results:**

Both SNEDDSs had lower and more uniform particle size distribution than C-CsA, and a similar optical density to phosphate-buffered saline and stable pH, in contrast to the high turbidity and unstable pH of C-CsA. Aqueous tear volume and fluorescein staining scores were improved in C-CsA- and SNEDDS-treated mice. Numbers of PAS-positive goblet cells and levels of inflammatory mediators were decreased by both C-CsA and SNEDDS, although SNEDDS resolved inflammation more effectively than C-CsA.

**Conclusions:**

Cyclosporine A eyedrops with SNEDDS have improved physicochemical properties and treatment efficacy in BAC-induced DED.

## Introduction

Dry eye disease (DED) is a multifactorial disorder of the ocular surface defined by dysfunctional tear film homeostasis [1]. Hyperosmolarity, tear film instability, neurosensory abnormalities, ocular surface inflammation and tissue damage contribute to the undesirable symptoms and aetiology of DED [1]. Ocular surface inflammation plays a significant role in DED pathology, and is primarily mediated by CD4^+^ T cells [2, 3]. Numerous inflammatory cytokines associated with conjunctival T cells are elevated in the tear film of DED patients [4]. Induction of DED-associated inflammation occurs due to a diverse array of pathologies. Systemic autoimmune diseases such as Sjögren syndrome lead to destruction of the lacrimal gland, causing aqueous deficient dry eye [1, 5]. Contrastingly, meibomian gland dysfunction diminishes tear film lipid content, resulting in evaporative dry eye [1, 5]. However, regardless of the cause, the downstream result is loss of tear film integrity and stability, promoting ocular surface inflammation and damage to the corneal epithelium. Therefore, ophthalmic anti-inflammatory agents are important treatments for all forms of DED.

Cyclosporine A (CsA) is the anti-inflammatory treatment of choice for DED, as it can be utilised long-term without adverse side effects associated with long-term use of other anti-inflammatory drugs such as corticosteroids [6]. In addition, unlike steroids, the specific and reversible effects of CsA on T cells make CsA desirable for extended use [7]. However, CsA has a large molecular weight and hydrophobicity (Log P = 1.4–3.0; solvent-dependent), resulting in poor aqueous solubility (6.6–106 lg/mL; temperature-dependent) [8]. Therefore, CsA is challenging to administer with conventional topical ophthalmic delivery systems. Thus, it is important to improve drug delivery strategies for CsA to maximise its ocular bioavailability. Over the past several decades, various drug delivery tactics have been developed to improve the ophthalmic bioavailability of CsA after topical administration to alleviate DED without the systemic side effects associated with oral CsA treatment [9]. These delivery strategies include hydrogels, *in situ* gelling systems, liposomes, nanoparticles and micelles [10]. Restasis (CsA ophthalmic emulsion 0.05%; Allergan, Irvine, CA, USA) is the most authorised and widely used CsA worldwide, and is a preservative-free, anionic oil-in-water emulsion, in which CsA is enclosed and dissolved in castor oil with the emulsifying agent polysorbate 80 [11]. However, Restasis is often associated with adverse effects such as visual disturbances due to its turbidity, ocular discomfort and conjunctival hyperaemia. Restasis is an anisotropic complex emulsion, which can break down when applied to the tear film, causing release of free surfactants that may irritate the ocular surface [12]. Also, in the dispersed phase, a wide range of particle sizes and distribution can cause creaming and flocculation of the preparation, decreasing its long-term physicochemical stability [13, 14]. Furthermore, Restasis is a complex emulsion, so CsA exists in various phases that may have differential biological effects, which may restrict sufficient delivery of bioactive CsA to the tissue [12].

On the other hand, 0.05% CsA nanoemulsion products based on the self-nanoemulsifying drug delivery system (SNEDDS) [15], such as Cyporin-N (SNEDDS-N; Taejoon Pharma, Seoul, South Korea) and T-sporin (SNEDDS-T; Hanlim Pharma, Seoul, South Korea), are currently available. In addition to cyclosporine, a significant number of other drugs have poor water solubility and permeability, leading to poor bioavailability and variable drug efficacy. SNEDDSs are anhydrous homogenous mixtures formed by mixing an oil, a drug, a surfactant, and a co-surfactant. Gentle dilution with water results in optically transparent emulsions, in which the sizes of the particles are a few hundreds of nanometers in diameter. SNEDDS result in better chemical and physical stability of drugs and higher solubility of water insoluble drugs, such as cyclosporine A, than conventional nanoemulsions [16] In one previous clinical study, the SNEDDS eyedrop had comparable effects to Restasis, with more rapid improvement of temporal conjunctival surface damage and tear film stability than Restasis [13].

These studies suggest that enhancing the bioavailability of CsA to ocular tissues is necessary to maximise the clinical potency of CsA for DED [9]. We hypothesised that the physicochemical stability provided by the homogenous and smaller particle size in SNEDDS eyedrops might increase the bioavailability of CsA to ocular tissues, especially the cornea and conjunctiva. In the present study, we compared the physicochemical characteristics and *in vivo* anti-inflammatory efficacy of three commercially available ocular CsA preparations, conventional 0.05% nanoemulsion CsA (Restasis, C-CsA) and two 0.05% nanoemulsion CsA preparations (SNEDDS-N and -T), in the murine benzalkonium chloride (BAC)-induced experimental DED model.

## Materials and methods

### Size distribution of CsA emulsions

In a pilot experiment, a significant discrepancy in particle size was identified between C-CsA and SNEDDS preparations. C-CsA preparations have highly variable particle sizes, with size ranging from several nanometers to several tens of micrometers. Therefore, two different types of particle analysers with different thresholds were used to accommodate each preparation. To evaluate all three CsA preparations, a liquid particle size analyser (Cilas 1064; Cilas, Orléans, France) was used to accommodate the wide range of size distribution in C-CsA. A laser nanoparticle analyser (Zetasizer Nano; Malvern Panalytical Ltd., Worcestershire, England) was also used to analyse SNEDDS-N and -T for a more sensitive analysis. Particle size analysis was performed at 25°C.

### Scanning electron microscope (SEM) evaluation of CsA emulsions

A drop of each 0.05% CsA emulsion was applied to an electron microscope (EM) sample stand and dried in an EM oven at 37°C for 12 h. The sample surface was coated with Platinum-Palladium (10 nm thickness) to reduce fluorescence. Each CsA emulsion was evaluated by SEM (Hitachi S-4200; SEMTech Solutions, North Billerica, MA, USA) at 20.0 kV.

### Turbidity of CsA emulsions

To evaluate CsA solubility in each preparation, light scatter was analysed at specific wavelengths using a microplate absorbance reader (iMark; Bio-Rad Laboratories, Hercules, CA, USA). After brief vortexing, 50 μL of each emulsion was placed in a 96-well plate. Absorbance was measured at 415, 450, 490, 560, 595, 655 and 750 nm. Phosphate-buffered saline (PBS), ultrapure water and balanced salt solution were used as references. Each measurement was repeated in triplicate and performed at 25°C.

### Instability index of CsA emulsions

Because the C-CsA emulsion is an unstable oil-in-water emulsion, phase separation may occur gradually over time. Therefore, the instability index was evaluated and compared over time for each CsA preparation. During a 2400 min experiment, the instability of each emulsion was detected by centrifugation at 2300g at 25°C or 35°C, and subsequent evaluation with a particle analyser (LUMiSizer; LUM GmbH, Berlin, Germany). Each value was detected every 3 min.

### pH instability of CsA emulsions

Since a changing pH value indicates unstable dissolution of the solute in the solvent, the stability of the emulsions can be indirectly determined by performing pH measurements. Unstable pH can also result in inconsistent drug efficacy and discomfort to the patient during administration of the drug. [17] Therefore, drug pH was monitored 30 min after brief vortexing at 25°C and 35°C using a benchtop pH meter (Orion Star A211; Thermo Fisher Scientific, Waltham, MA, USA). The pH value of each CsA emulsion was read every minute.

### Static and kinematic viscosity

Because eyedrop viscosity affects drug efficacy and blurring after instillation, and nanoemulsification decreases viscosity, we measured dynamic (absolute) viscosity using a Brookfield type viscometer (Brookfield DV2T, SC4-18, 5 rpm, 25°C), and kinetic viscosity using the following equation: Kinematic viscosity = K (0.08390) x t (mean value of three repeated measures using a capillary tube viscometer).

### Induction of murine BAC-induced DED model and application of CsA emulsion eyedrops

All study procedures were conducted in accordance with the ARVO Statement for the Use of Animals in Ophthalmic and Vision Research. The animal protocol was approved by the Keimyung University Institutional Animal Care and Use Committee (KM-2018-11R).

Ninety 8-week-old male C57BL6J mice were purchased from Hyochang Science (Daegu, South Korea). Mice were kept in a facility with a standard environment (temperature, 25°C ± 1°C; relative humidity, 60% ± 5%; and 12 h light/12 h dark cycles [9 AM to 9 PM]), and provided food and water *ad libitum*. Experimental dry eye was induced by twice-daily (9 AM and 6 PM) application of 5 μL of 0.2% BAC (Sigma-Aldrich) for 2 weeks. After BAC administration was complete and DED was successfully induced, mice were randomly divided into six groups (15 mice per group): Naïve control, non-treated control, BSS-treated control, C-CsA, SNEDDS-N and SNEDDS-T groups. Fifteen naïve control mice without BAC-induced DED were used as a normal control.

After group allocations, the naïve control was maintained for a 4 week experimental period (untreated for 2 week DED induction period and 2 week DED treatment period), the untreated control group was maintained without application of eyedrops for 2 weeks after the 2 week BAC induction period, the solvent-treated control was treated with balanced salt solution (BSS; Alcon Laboratories, Fort Worth, TX, USA) twice daily for 2 weeks after the 2 week DED induction period and the three 0.05% CsA treatment groups were treated twice daily (9 AM/6 PM) with the specified CsA preparation for 2 weeks after the 2 week DED induction period (Fig 3A). At 1 and 2 weeks of dry eye treatment, 2, 2, 2-tribromoethanol (Avertin) was injected intraperitoneally at a 250 mg/kg dose for evaluations of the corneal fluorescein staining and tear meniscometry. Two weeks after dry eye treatment, mice were sacrificed through cervical dislocation and the eyeballs were enucleated and analyzed.

### Corneal fluorescein staining and tear meniscometry

At both 1 and 2 weeks after beginning treatment of DED animals, corneal fluorescein staining scoring and tear volume measurement were performed. After application of fluorescein dye using fluorescein paper (Haag-Streit AG, Koeniz, Switzerland) into the lower conjunctival sac, corneal staining scores were calculated under cobalt blue light using a portable slit lamp (SL-17; Kowa Optimed, Torrance, CA, USA). The corneal staining score was calculated by a blinded investigator (C.Y.Y.) as follows: absent, 0; slightly punctuate staining with fewer than 30 spots, 1; non-diffuse punctuate staining with more than 30 spots, 2; severe diffuse staining with no positive plaques, 3; and positive fluorescein plaques, 4. The scores from four quadrants were averaged to a final grade (ranging from 0 to 16 points) (Fig 3B). Tear volume was measured 1 and 2 weeks after beginning CsA treatment using strip meniscometry (SM Tube; EchoElectricity Co., Ltd., Fukushima, Japan) from the lower conjunctival sac. After anaesthesia, the tear meniscometry tip was introduced to the lower tear lake for 5 s. The length of wetted thread was measured per manufacturer’s instructions.

### Immunohistochemistry of central and peripheral cornea

The eye and ocular adnexa were excised, fixed in 4% paraformaldehyde for 3 days and embedded in optimal cutting temperature (OCT) compound at -80°C. A 7 μm section was cut and air dried at room temperature. Sectioned tissues were permeabilised with 0.4% (v/v) Triton X-100 (Structure Probe, West Chester, PA, USA) for 15 min and blocked with 1% (w/v) bovine serum albumin in 0.1% (v/v) Tween 20 (AMRESCO, Solon, OH, USA) in PBS for 30 min. Sections were then incubated with anti-IL-1β, IL-6, TNF-α, CK-10 and Ki-67 primary antibodies (BioLegend, San Diego, CA, USA) overnight at 4°C, and subsequently with Alexa Fluor 594-conjugated secondary antibodies (Molecular Probes, Eugene, OR, USA) for 2 h at room temperature. After several rinses, nuclei were stained with Vectashield mounting solution (Vector Laboratories, Burlingame, CA, USA) containing 4',6-diamidino-2-phenylindole (DAPI). Each slide was observed under an upright fluorescence microscope (Leica Microsystems GmbH, Wetzlar, Germany), and images were obtained using an inverted fluorescence microscope (Ti-U; Nikon Instruments, Melville, NY, USA), and captured using NIS-Elements software (Nikon Instruments). Ki-67-positive cells at the basal area of the corneal epithelium were counted with ImageJ (http://imagej.nih.gov/ij/; provided in the public domain by the National Institutes of Health, Bethesda, MD, USA) at five different locations by a blinded investigator (C.Y.Y.). All experiments were performed in triplicate.

### TUNEL assay

To compare the treatment efficacy of each CsA preparation, TUNEL staining was performed on corneal cryosections using an *in situ* cell death detection kit (Merck, Kenil Worth, NJ, USA) per the manufacturer’s instructions. Each section was counterstained and mounted with Vectashield mounting medium (Vector Laboratories). Each slide was examined, and images were captured as described for immunohistochemistry.

### PAS staining

Eyeballs and adnexa were surgically excised and fixed with 4% paraformaldehyde (Biosesang, Seongnam-si, Gyeonggi-do, South Korea) for 3 days at 4°C followed by a 3 day PBS (Welgene, Gyeongsan-si, Gyeongsangbuk-do, South Korea) rinse, cryoprotected using a sucrose gradient and embedded in OCT compound (Tissue-Tek; Sakura Finetek, Torrance, CA, USA) at -80°C. A 7 μm section was cut and dried at room temperature. The sectioned tissue was stained with periodic acid-Schiff (PAS; Sigma-Aldrich) for 15 min, with haematoxylin counterstaining (Muto Pure Chemicals, Tokyo, Japan) for 5 min. Each slide was examined and photographed under a microscope (Ti-U; Nikon Instruments), and images were captured using NIS-Elements software (Nikon Instruments). PAS-positive goblet cells were counted with ImageJ on three individual slides for each treatment by a blinded investigator (C.Y.Y.).

### RNA isolation and real-time polymerase chain reaction

Total corneal and conjunctival RNA was extracted using a NucleoSpin RNA Plus kit (Macherey-Nagel GmbH & Co. KG, Düren, Germany), and cDNA was synthesised using ReverTraAce qPCR RT Master Mix (FSQ-201; TOYOBO, Osaka, Japan). Real-time PCR was performed using a CFX96 real-time system (Bio-Rad Laboratories) using SsoAdvanced Universal SYBR Green Supermix (172–5271; Bio-Rad Laboratories). Primer sequences are shown in Table 1. The amplification program included an initial denaturation step at 95°C for 30 s, followed by 40 cycles of 95°C for 15 s and 60°C for 30 s, after which a melt curve analysis was conducted to check amplification specificity. Results were analysed by the comparative threshold cycle (Ct) method, normalised with HPRT as an endogenous reference and calibrated against the normal control group.

**Table 1:** Primer sequences for semi-quantitative real-time PCR.

Gene	Accession no.	Sequence (5'→3')	Product size (bp)
ICAM-1	NM_010493.3	Forward: TTCTCATGCCGCACAGAACT	73
		Reverse: TCCTGGCCTCGGAGACATTA	
VCAM-1	NM_011693.3	Forward: CTGGGAAGCTGGAACGAAGT	115
		Reverse: GCCAAACACTTGACCGTGAC	
F4/80	NM_010130.4	Forward: GTGCCATCATTGCGGGATTC	79
		Reverse: AAGAGCATCACTGCCTCCAC	
MMP-2	NM_008610.3	Forward: CTGATGGCCCCGATCTACAC	74
		Reverse: AGCTCCTGGATCCCCTTGAT	
HPRT	NM_013556.2	Forward: CCTAAGATGAGCGCAAGTTG	86
		Reverse: CACAGGACTAGAACACCTGCTAA	

### Western immunoblot analysis

Cornea and conjunctival tissues were excised. Frozen cornea and conjunctival tissues were crushed with stainless beads in a bead mill homogeniser (Bullet blender; Next Advance, Troy, NY, USA). Samples were then lysed in radioimmunoprecipitate buffer (Elpis Biotech, Daejeon, South Korea). Lysate protein concentration was measured using a protein bicinchoninic acid assay kit (Thermo Fisher Scientific). Equal quantities of total protein samples were loaded onto 8–15% polyacrylamide gels for sodium dodecyl sulphate polyacrylamide gel electrophoresis (Bio-Rad Laboratories). Resolved proteins were electroblotted onto nitrocellulose blotting membranes (Sigma-Aldrich) and blocked with 5% skim milk solution (Honeywell International, Morris Plains, NJ, USA) for 2 h. After overnight incubation with the appropriate primary antibodies, the membranes were incubated with secondary antibodies for 1 h. The fluorescent band of a stain-free gel (Mini-PROTEAN TGX stain-free gel; Bio-Rad Laboratories) was used as a loading control for band densitometry.

### Statistical analysis

Representative data obtained from experiments conducted in triplicate are presented as mean ± standard deviation. Data were evaluated using independent t-tests for two groups, or one-way analysis of variance (ANOVA) for three or more groups and Tukey’s post hoc comparison, to identify statistically significant differences. A p-value <0.05 was considered statistically significant.

## Results

### SNEDDS preparations reduced particle size, particle size variability, preparation turbidity, and viscosity

**Fig 1:**
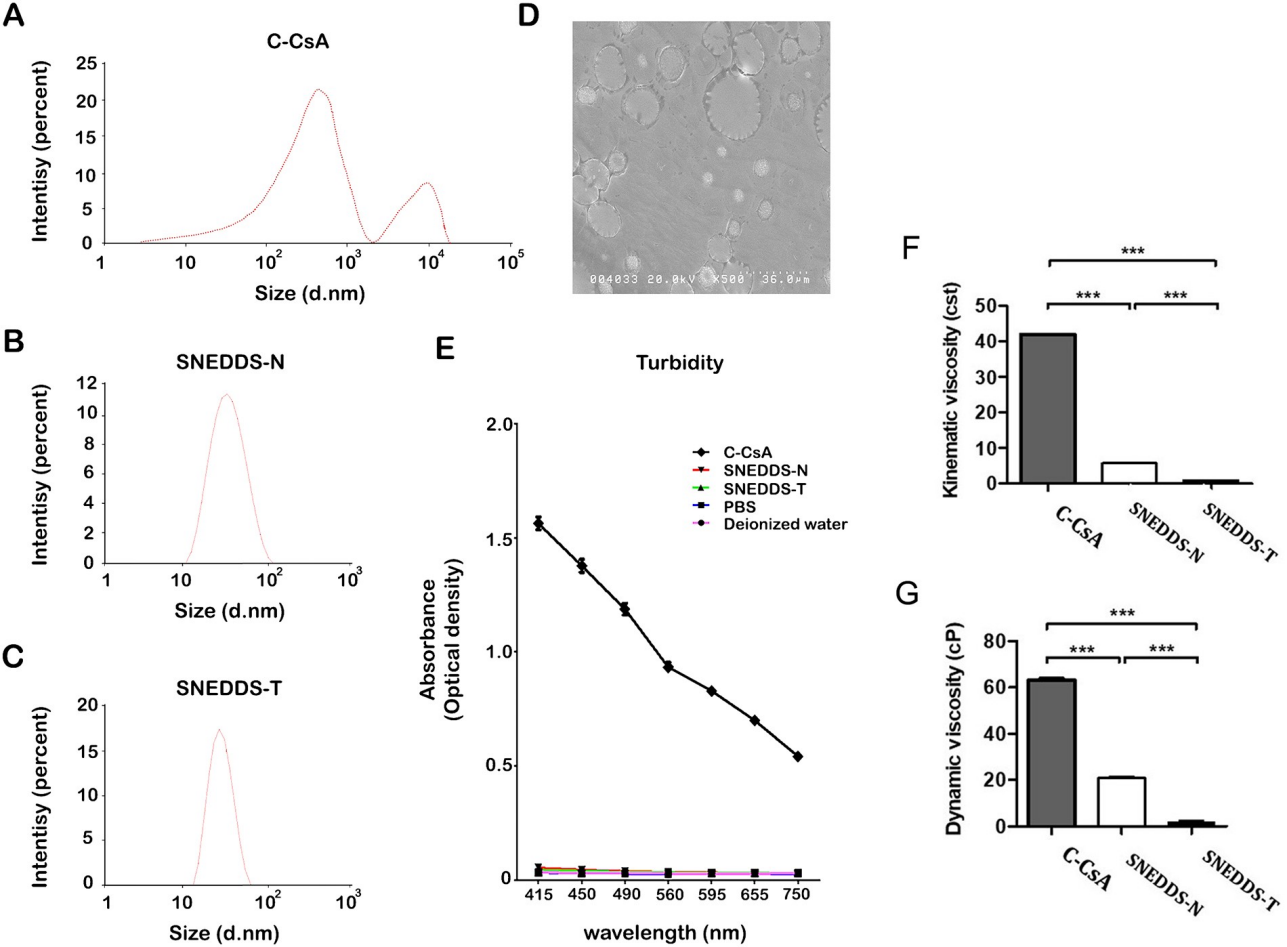
Size distribution (percent) of CsA emulsions by intensity: (A) Restasis (C-CsA), (B) T-sporin (SNEDDS-T) and (C) Cyporin-N (SNEDDS-N). (D) Scanning electron microscope (SEM) evaluation of C-CsA. (E) Turbidity (optical density) of CsA emulsions.

Based on particle size analysis, self-nanoemulsified CsA particles were distributed evenly and in a single size. Particle size analysis of C-CsA identified two larger size peaks at approximately 1000 and 10,000 nanometres with a wider range (6–13,000 nm) (Fig 1A). However, both SNEDDS preparations had a single peak in particle distribution (SNEDDS-N: 42.67 ± 20.59 nm, SNEDDS-T: 26.49 ± 8.32 nm) (Fig 1B and 1C). SEM analysis revealed variable particle sizes in C-CsA (Fig 1D); however, particles of the SNEDDS preparations were not identifiable by the general sample preparation technique.

C-CsA is a milky colour emulsion due to a mixture of unevenly distributed, variably sized oil-in-water emulsions. Contrastingly, an evenly distributed and single-size emulsion would have improved transparency. Therefore, we evaluated the effects of self-nanoemulsification on CsA preparation turbidity. C-CsA had much higher turbidity than PBS or BSS at every wavelength (Fig 1E). However, both SNEDDS preparations had extremely low turbidity, which was similar to that of ultrapure water, PBS and BSS (Fig 1E). Both SNEDDS-T and SNEDDS-N showed significantly lower turbidity than C-CsA (Fig 1F and 1G).

### SNEDDS preparations had lower instability indices and stable pH

**Fig 2:**
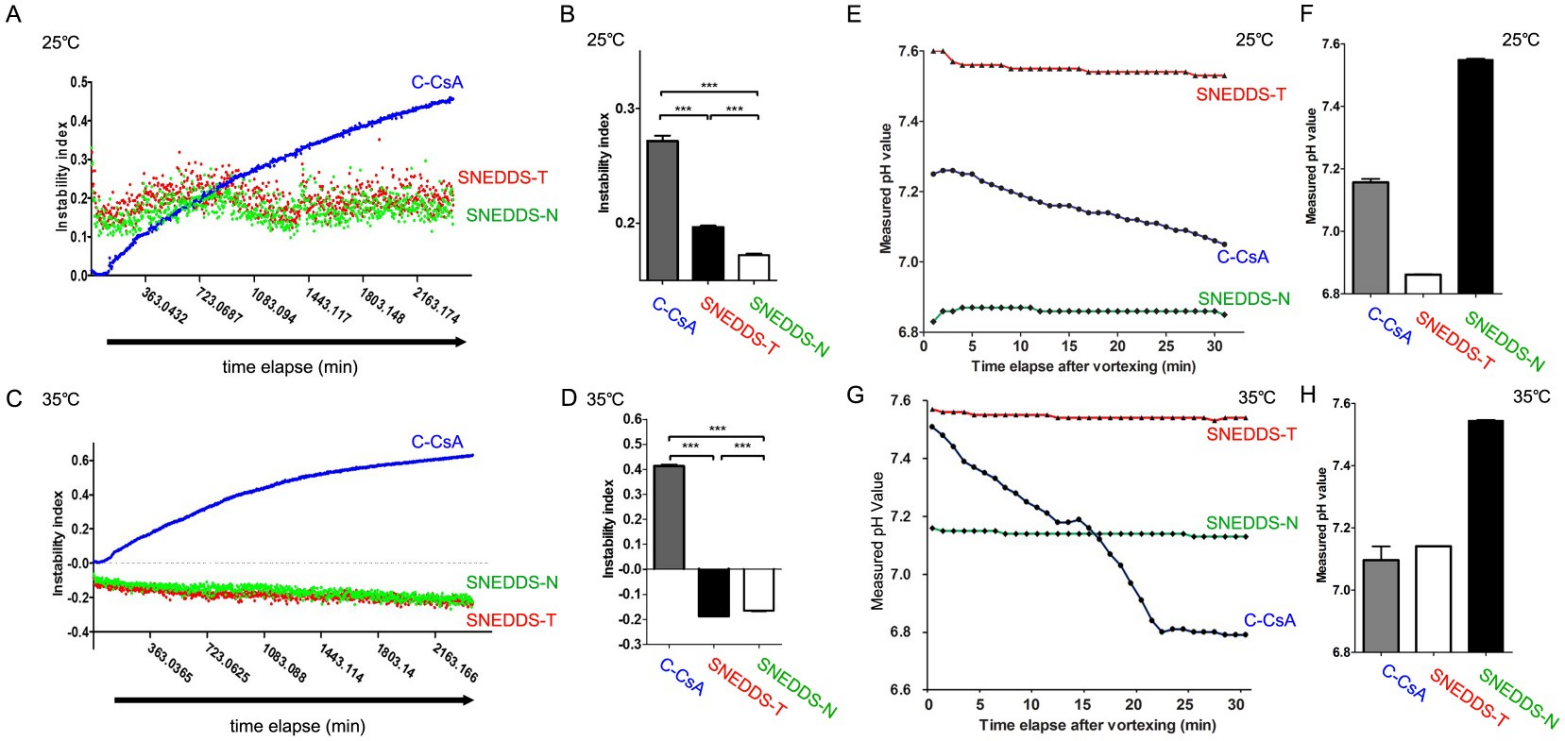
(A) Instability index along time elapse (2400 min) and (B) mean instability index of CsA emulsions (n = 30). (C) pH instability along time elapse after vortexing (30 min) and (D) mean pH values of CsA emulsions (n = 30). Data are expressed as mean ± SD. *P < 0.05, **P < 0.01, ***P < 0.001 by independent t-test.

Because C-CsA is manufactured using a high-speed homogeniser, the drug itself is unstable, and is likely to be denatured over time due to flocculation and creaming. Contrastingly, the SNEDDS preparations are likely to be more stable, as these preparations are self-emulsifying. Therefore, we analysed the instability indices of the three CsA preparations. Both SNEDDS preparations were remarkably stable until 2400 min, but C-CsA exhibited a gradual increase in instability index at 25°C and 35°C (Fig 2A–2D). In addition, consistent with the instability results, the pH of each preparation exhibited a similar pattern. After brief vortexing of each preparation, C-CsA exhibited a gradual decrease in pH over 30 min, while the pH of the SNEDDS preparations remained stable at 25°C and 35°C (Fig 2E–2H).

### SNEDDS preparations conferred earlier recovery of corneal fluorescein stain and tear volume in the murine DED model

**Fig 3:**
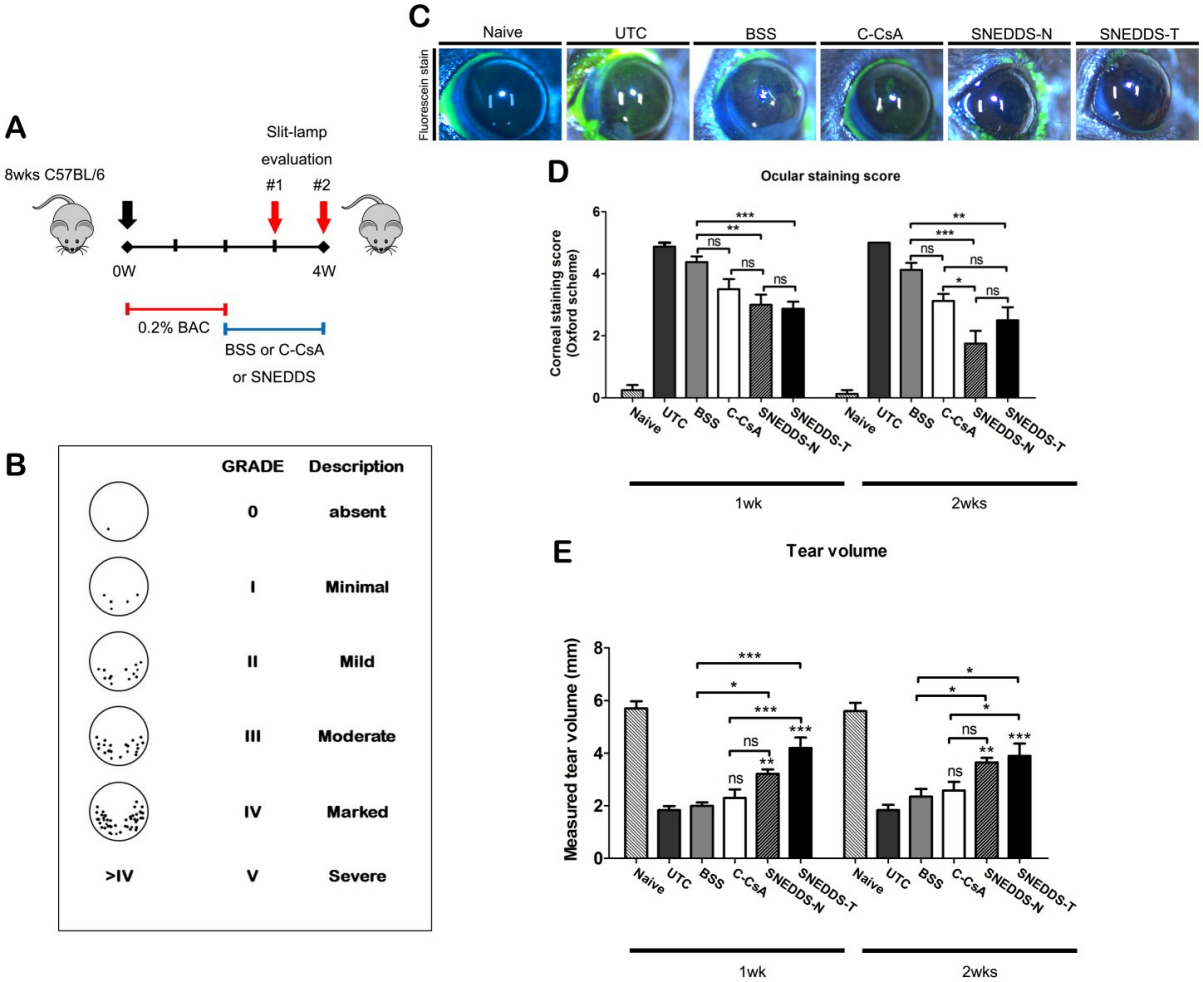
(A) Schematic diagram of experimental protocol of the murine benzalkonium chloride (BAC)-induced DED model and subgroups. (B) Schematic diagram of corneal fluorescein staining scoring system. (C) Representative images of corneal fluorescein staining in the untreated control (UTC), balanced salt solution (BSS)-treated, C-CsA-treated, SNEDDS-N-treated and SNEDDS-T-treated subgroups on week 2. (D, E) *In vivo* evaluations of dry eye murine model by (D) corneal fluorescein staining (n = 8) and (E) tear volume (n = 8) on weeks 1 and 2. Data are expressed as mean ± SD. *P < 0.05, **P < 0.01, ***P < 0.001 by independent t-test or one-way ANOVA.

After 2-week induction of experimental dry eye using 0.2% BAC, we monitored alterations in corneal fluorescein staining scores at 1 and 2 weeks after treatment with each preparation. Both SNEDDS preparations conferred accelerated recovery of corneal staining relative to the BSS and untreated control groups (Fig 3C and 3D). Although all three CsA eyedrops improved the staining score compared with the BSS or untreated control group at 1 week after initiation of treatment, SNEDDS preparations had similar or higher treatment efficacy than C-CsA (Fig 3C and 3D). C-CsA did not significantly improve tear volume at 1 and 2 weeks after treatment, but the SNEDDS preparations significantly improved tear volume at both 1 and 2 weeks (Fig 3E).

### SNEDDS preparations ameliorate corneal epithelial inflammation and squamous metaplasia

**Fig 4:**
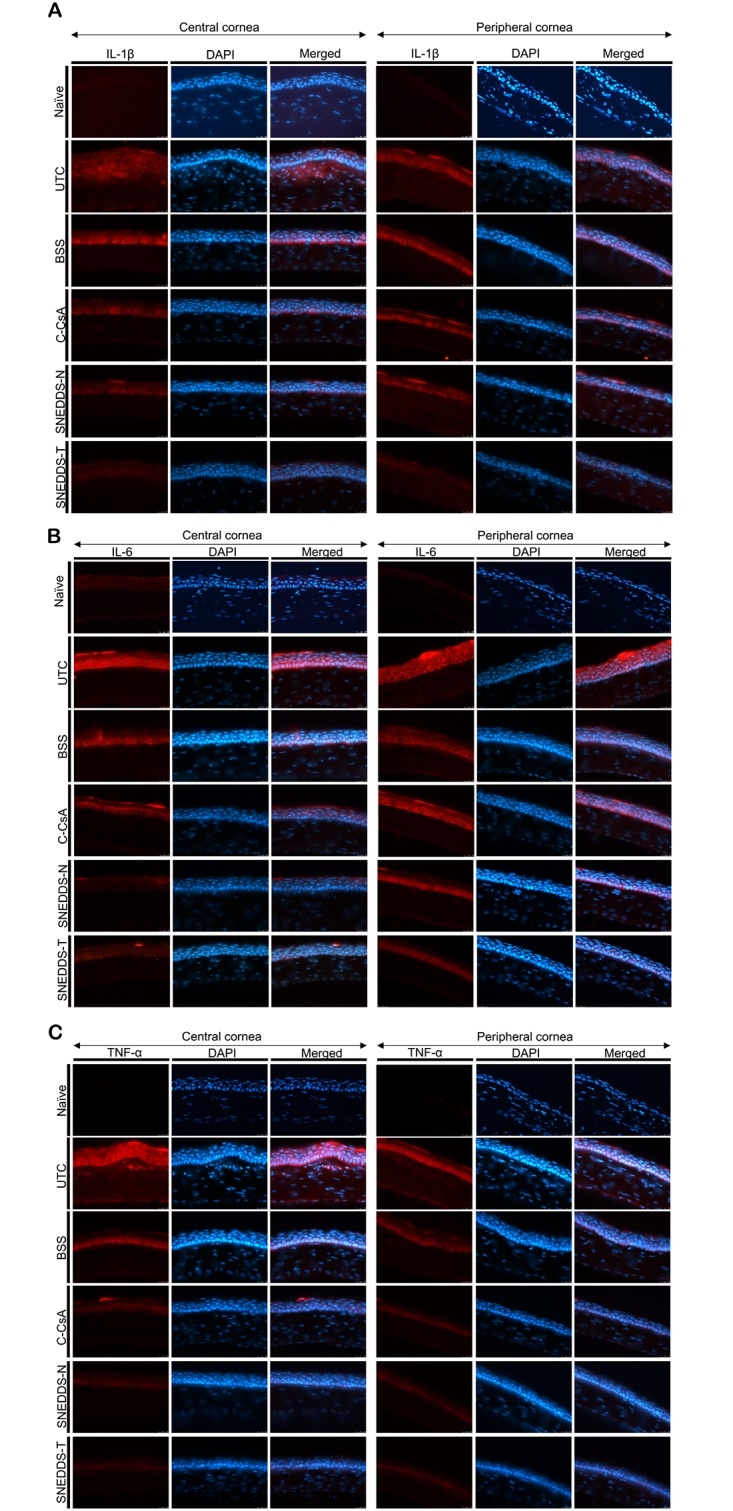
Representative images for immunofluorescent staining of inflammatory cytokines including (A) IL-1β, (B) IL-6 and (C) TNF-α in central and peripheral cornea in the UTC, BSS-treated, C-CsA-treated, SNEDDS-N-treated and SNEDDS-T-treated subgroups.

**Fig 5:**
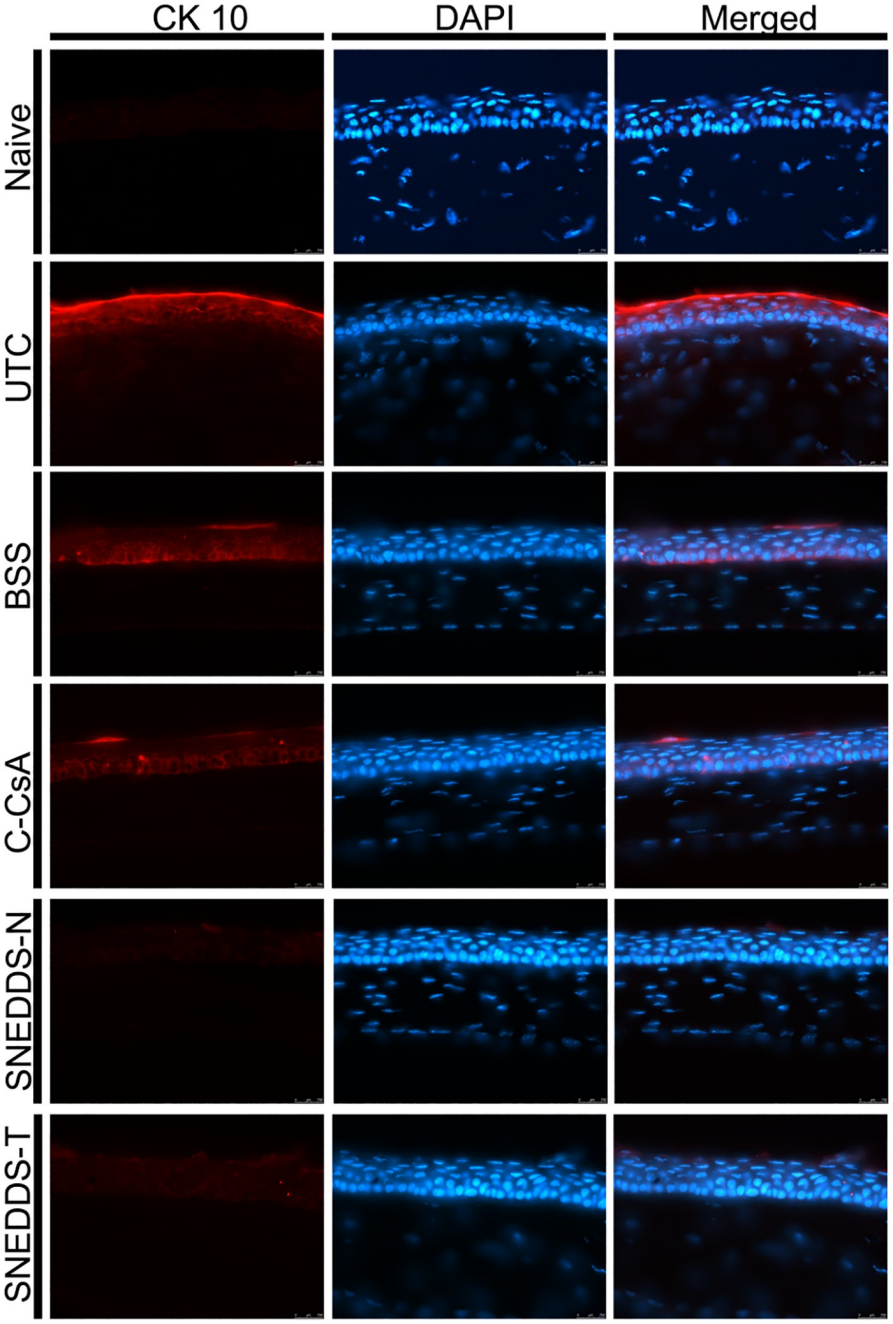
Representative images for immunofluorescent staining of CK-10 in central cornea to assess corneal epithelial squamous metaplasia in the untreated control, BSS-treated, C-CsA-treated, SNEDDS-N-treated and SNEDDS-T-treated subgroups.

After 2 week application of each eyedrop, we enucleated the eyes of all animals, and measured levels of inflammatory mediators in both the central and peripheral cornea using IHC. Both SNEDDS preparations decreased levels of IL-1β, IL-6 and TNF-α relative to both the BSS and C-CsA groups (Fig 4). In addition, we assessed corneal surface metaplasia of each group using anti-CK-10 immunostaining. Corneal squamous metaplasia was almost completely recovered in SNEDDS-treated animals, while metaplasia was only partially recovered by C-CsA treatment (Fig 5).

### SNEDDS preparations increased Ki-67 staining and decreased numbers of apoptotic cells in the corneal epithelium

**Fig 6:**
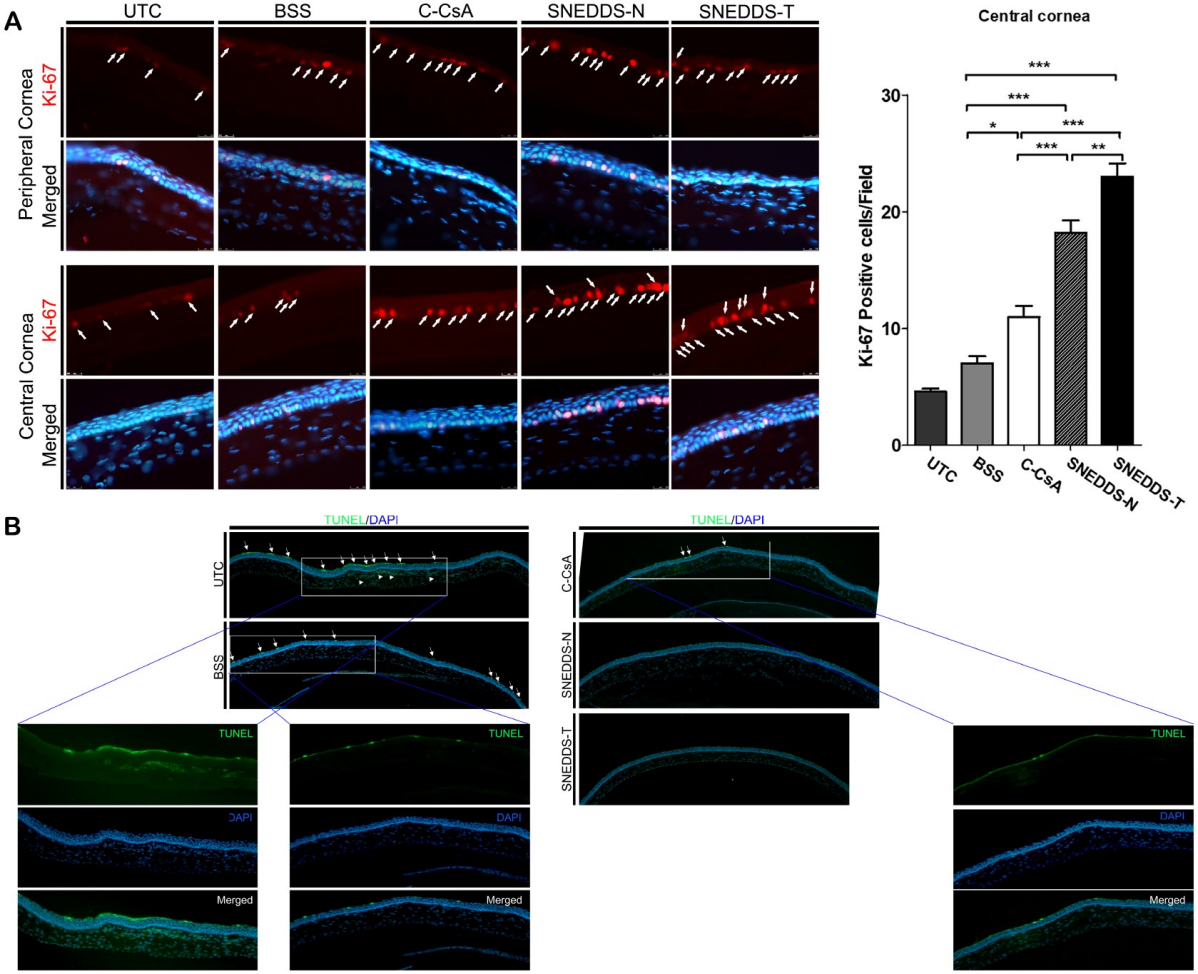
Representative images for (A) immunofluorescent staining of Ki-67 in central (n = 5) and peripheral cornea to assess corneal epithelial cell proliferation, and (B) TUNEL assay to evaluate corneal epithelial cell apoptosis in the untreated control, BSS-treated, C-CsA-treated, SNEDDS-N-treated and SNEDDS-T-treated subgroups. Data are expressed as mean ± SD. *P < 0.05, **P < 0.01, ***P < 0.001 by independent t-test.

We evaluated the effect of each CsA preparation on corneal epithelial proliferation and apoptosis. Expression of Ki-67 was increased in both the central and peripheral cornea in all CsA groups relative to the BSS-treated control, which was suggestive of increased cellular proliferation (Fig 6A). However, SNEDDS-treated animals had more Ki-67^+^ cells than did C-CsA-treated animals (Fig 6A). Additionally, we utilised a TUNEL assay to investigate DED-induced corneal epithelial apoptosis. C-CsA decreased apoptotic superficial corneal epithelial cells relative to the BSS control, but both SNEDDS preparations further decreased apoptosis relative to C-CsA, with very few apoptotic cells detected (Fig 6B).

### Effect of SNEDDS preparations on goblet cell density

**Fig 7:**
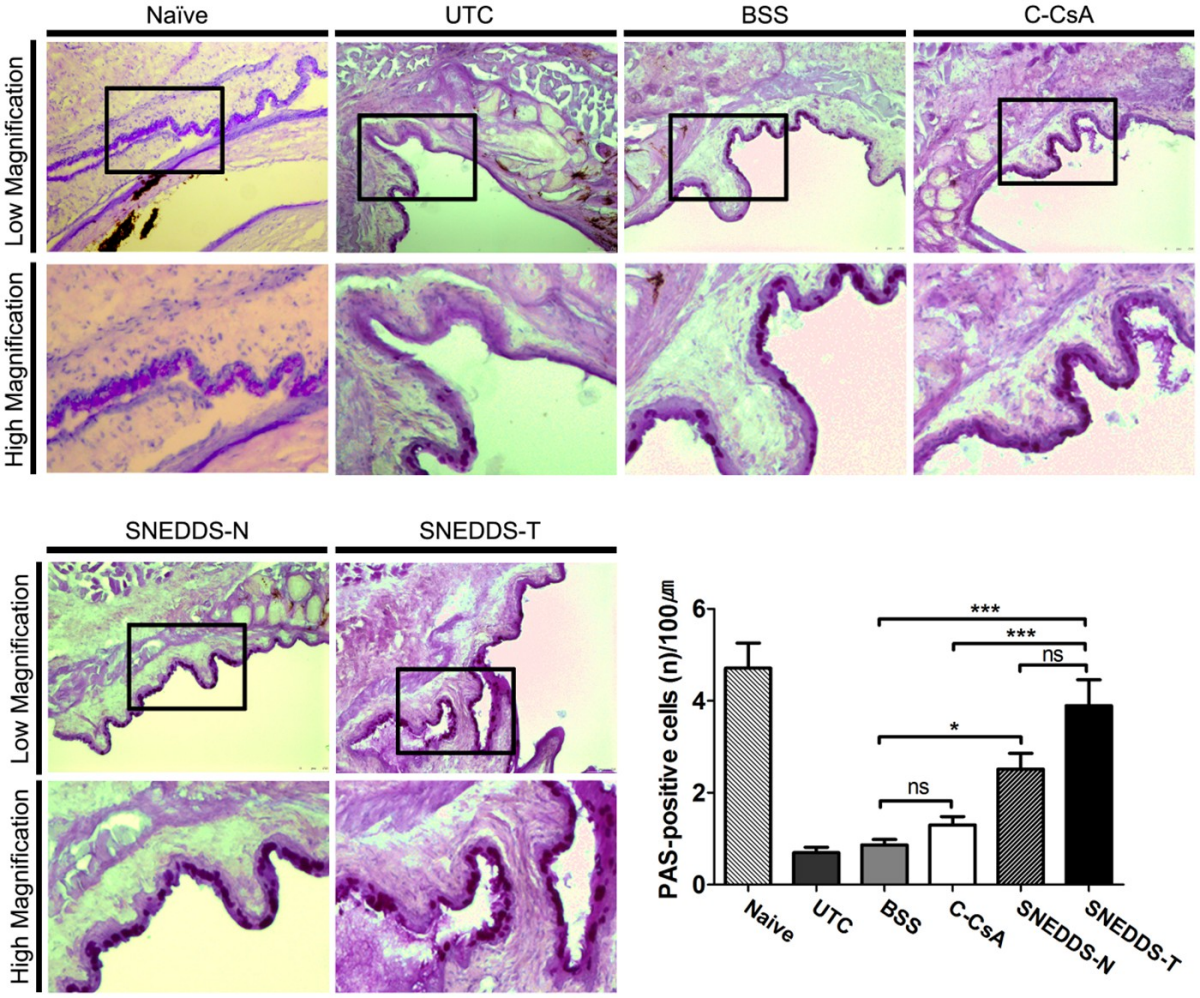
Mucin-containing goblet cells along the conjunctival fornices were visualised by PAS staining in the untreated control, BSS-treated, C-CsA-treated, SNEDDS-N-treated and SNEDDS-T-treated subgroups, and mean numbers of PAS^+^ cells in each subgroup were quantified (n = 5). Data are expressed as mean ± SD. *P < 0.05, **P < 0.01, ***P < 0.001 by independent t-test.

We quantified goblet cell density in DED animals treated with each CsA preparation using PAS staining. Both SNEDDS preparations increased goblet cell density at the 2 week timepoint with greater efficacy than either C-CsA or the BSS control, while C-CsA did not show significant superiority in efficacy to the BSS control (Fig 7).

### Effect of SNEDDS preparations on corneal and conjunctival surface inflammation

**Fig 8:**
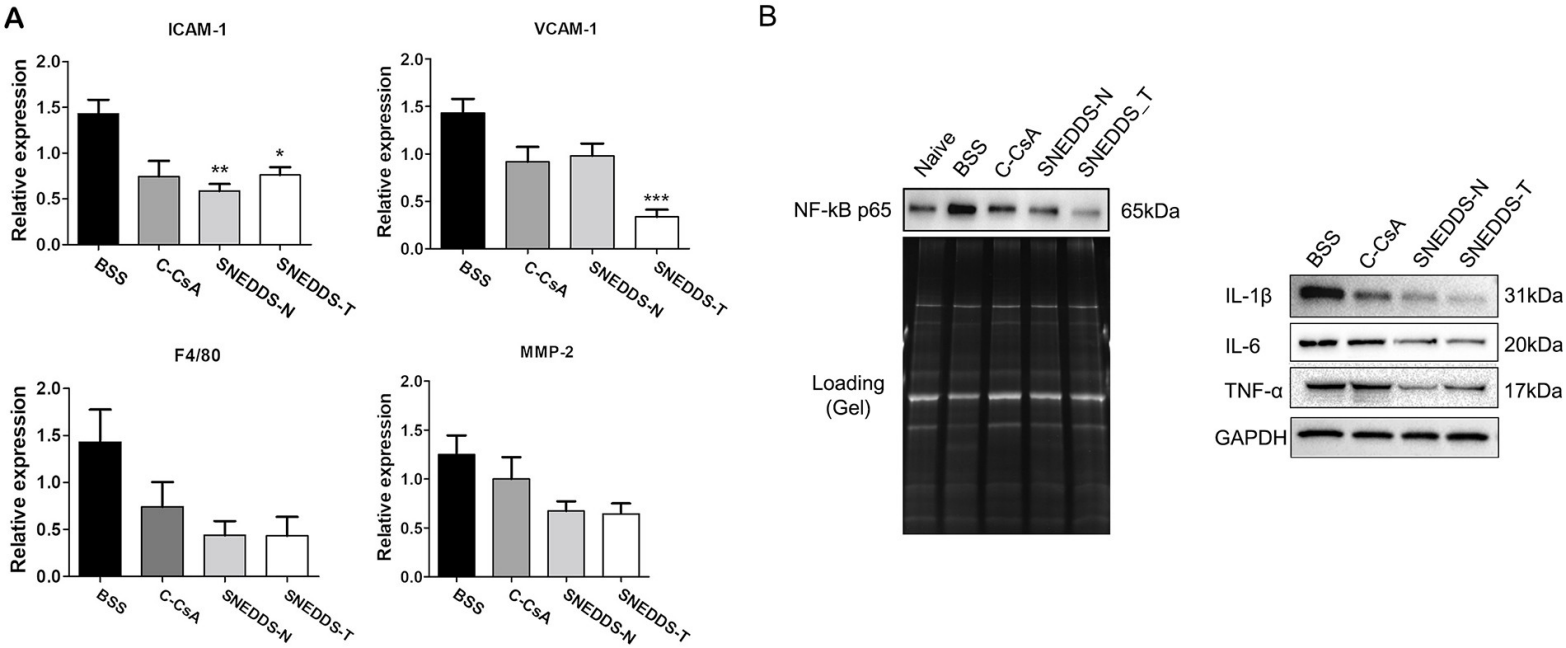
(A) Real-time PCR was used to quantify corneal mRNA expression of *Icam-1*, *Vcam-1*, *F4/80* and *Mmp-2* in each subgroup (n = 5). (B) Corneal NF-κB activation and IL-1β, IL-6 and TNF-α expression were assessed by western blot analysis (n = 5). Data are expressed as mean ± SD. *P < 0.05, **P < 0.01, ***P < 0.001 by independent t-test.

To compare and verify the regulatory effects of topical CsA, we measured mRNA and protein levels of inflammatory mediators in the corneoconjunctival tissue. Both SNEDDS preparations significantly decreased ICAM-1 and VCAM-1 mRNA levels relative to the BSS control, suggesting that they decreased inflammation more effectively (Fig 8A). In addition, both SNEDDS preparations decreased NF-κB activation and protein levels of 1L-1β, IL-6 and TNF-α relative to the BSS control and C-CsA, as demonstrated by the results of western blot analysis (Fig 8B).

## Discussion

An emulsion is a liquid-liquid dispersion system in which at least one liquid is dispersed in another liquid with which it is immiscible. Most emulsions have a particle size distribution ranging from 0.1 to several tens of micrometers [18]. Microemulsions are thermodynamically unstable, and ultimately separate through various processes, including flocculation, sedimentation, creaming, Ostwald ripening, and coalescence [19]. If the sizes of dispersed emulsion particles are reduced to the nanometer range, emulsion stability is greatly enhanced due to Brownian motion between particles [20]. C-CsA is a nanoemulsion with a particle size of the dispersed phase in the nanometer range.

To prepare C-CsA as a nanoemulsion, a high-pressure homogeniser may be used to apply high physical power to the emulsion [21], or a high-speed stirring or shearing machine, such as a microfluidiser, may also be used [22]. These preparation methods are costly and require large preparation equipment. Further, these methods apply high energy to the emulsion, significantly increasing temperature during emulsification, which is detrimental to emulsion components vulnerable to heat, such as CsA, which is a phospholipid [23]. In addition, Restasis has non-uniform particle size, increasing flocculation and creaming, which makes long-term storage prohibitive [13]. Further, because the distribution of particles in the dispersed phase is relatively wide, it is difficult to ensure uniform product quality in every preparation lot [24]. On the other hand, SNEDDS is formulated without the use of high-pressure homogenisation, and has enhanced nanoemulsion stability due to homogeneity of particle size and distribution.

Due to the discrepancies in physiochemical properties between the C-CsA and SNEDDS preparations, we evaluated particle size distribution of each preparation with two different methods, including a laser particle size analyser and an ultrasound liquid particle analyser. This analysis demonstrated that the particle sizes of both SNEDDS preparations were distributed in a narrower range, while C-CsA exhibited an extremely wide size distribution. C-CsA is an anisotropic complex emulsion existing in multiple phases, which may cause the wider range of particle size [12]. CsA emulsified in C-CsA may have distinct bioactivities in each phase, and only a fractional portion of the CsA may penetrate corneal and conjunctival tissues to suppress inflammation.

In contrast to the conglomerate emulsions in C-CsA, SNEDDS is an isotropic liquid formulation of oil, water and surfactants. This Winsor type IV nanoemulsion consists of a single phase, such that the activity of CsA is consistent throughout the nanoemulsion. In the present study, these preparations were optically transparent with extremely low turbidity, and were thermodynamically and chemically stable, as demonstrated by low instability indices and consistent pH values over time. Unlike the high-energy preparation method for C-CsA, SNEDDS is formulated using a low-energy method [25]. The mixture of surfactant and cosurfactant at the appropriate ratio of oil and water cause a spontaneous formulation of the nanoemulsion, with a homogeneous droplet size retaining the internal phase. We detected different particle size distribution in the two SNEDDS preparations, which may be due to a differential composition of surfactant/cosurfactant.

A previous clinical comparative study demonstrated that another SNEDDS CsA preparation, Clacier, more rapidly improved tear breakup time scores than did C-CsA [13]. Additional prior studies demonstrated that the effects of topical C-CsA continue to improve with use, and that long-term topical CsA therapy alleviates disease progression [11, 26, 27]. We hypothesised that SNEDDS is a faster-acting agent due to increased CsA bioavailability afforded by the smaller homogenous particle size. In the present study, the SNEDDS preparations may have enhanced anti-inflammatory efficacy due in part to the relatively short course of treatment in the murine DED model. While there was a significant difference in tear film stability between the C-CsA and SNEDDS groups after 4 weeks of treatment in a previous clinical study [13], we demonstrated improved anti-inflammatory activity of SNEDDS even after 2 weeks of treatment. This suggests that enhancing the physicochemical properties of topical CsA by reducing particle size and improving homogeneity and stability can reduce the induction time of the agent.

BAC-induced dry eye causes pathologic changes of the corneal epithelium, including squamous metaplasia and apoptosis, which are closely associated with inflammatory progression [28]. We used this murine DED model to demonstrate that all three topical CsA preparations significantly decreased corneal fluorescein staining in DED, which suggests repair of ocular surface damage and improved corneal epithelium integrity, consistent with the results of a previous clinical study [13]. Interestingly, at 2 weeks of treatment, there was no significant difference in fluorescein staining between the BSS group and C-CsA group. This result indicates that even simple hydration of the cornea with BSS promotes corneal epithelial wound healing, potentially due to surface protection and humidity preservation, independent of anti-inflammatory effects.

The Schirmer tear test is a standard test for quantitative assessment of tear production, but is difficult to perform in rodent models because the test takes a long time (60 s for veterinary application) and causes irritation that is intolerable to mice. Strip meniscometry is a simple and fast method (5 s) for quantitative evaluation of tear volume [29], and can easily be conducted in rodent models. To evaluate the efficacy of topical CsA treatments, it is preferable to assess both lacrimal gland function and ocular surface integrity. Tear volume was increased by both SNEDDS preparations relative to BSS and C-CsA, consistent with previous reports [13, 30]. Together with the corneal staining result, this suggests that topical CsA treatment, especially in SNEDDS preparations, improves the epithelial quality of the corneal surface by ameliorating lacrimal gland inflammation. These improvements of the ocular surface and lacrimal gland secretion were likely related to tear film stability.

A previous study demonstrated that, in the murine BAC-induced DED model, corneal expression of the proinflammatory cytokines IL-1β, IL-6 and TNF-α and the chemokine MIP-2 was increased, suggesting inflammatory pathologies similar to those of human DED [31]. We demonstrated that all three topical CsA preparations, especially SNEDDSs, reduced expression of proinflammatory cytokines such as IL-1β, TNF-α and IL-6 and chemokines such as ICAM-1, VCAM-1. ICAM-1, synthesized by conjunctival epithelium cells, serves as a potent signalling molecule to induce ocular surface inflammation and lymphocytic infiltration of the lacrimal gland. In addition, the ocular surface adhesion molecule, VCAM, may induce ocular surface damage and recruit leukocytes along with ICAM-1 and E-selectin from the vascular compartment of the ocular surface. These results are consistent with those of previous studies, which identified CsA as an anti-inflammatory agent [32, 33].

Previous studies have reported that DED triggers p38 MAPK activation, which increases activity of NF-κB and levels of inflammatory mediators, including cytokines and MMPs [34–36]. CsA was reported to suppress p38 MAPK activation in an inflammatory model of DED [37]. The present study demonstrated that BAC induction of DED provoked NF-κB activation, which was markedly suppressed by topical CsA, consistent with decreased expression of inflammatory mediators. We suggest that CsA suppression of NF-κB signalling may down-regulate expression of inflammatory molecules, including cytokines such as IL-1β [38–40], TNF-α [40, 41] and IL-6 [40, 42, 43], and chemokines [44–46] such as ICAM-1 and VCAM-1.

Squamous metaplasia, in which corneal epithelial cells are transformed into keratinised cells, is associated with numerous ocular surface diseases, including DED [47]. Proinflammatory cytokines contribute to squamous metaplasia [48], and the severity of DED and intensity of squamous metaplasia are correlated in DED patients [49]. Cytokeratin-10 (CK-10) expression in the corneal epithelial layer is indicative of squamous metaplasia. In the present study, we found that CK-10 expression was increased after BAC induction of DED, and decreased by topical CsA treatment. This suggests that topical CsA treatment successfully suppresses scar formation after superficial punctate keratitis related to DED.

Ki-67 is indicative of cell cycle status, as Ki-67 is expressed during the G1, S, G2 and M phases, but not in the G0 phase, of the cell cycle [50]. Therefore, we measured Ki-67 expression in corneal epithelial cells to evaluate the effect of topical CsA on the corneal epithelial proliferation, and found that both SNEDDS preparations significantly increased proliferation. A previous study identified that BAC causes DNA damage in corneal epithelial cells, and decreases Ki-67 protein expression in human corneal cells [51]. Other previous studies using the murine BAC-induced DED model identified that few Ki-67^+^ cells were detected in the superonasal corneal epithelium in both untreated control and solvent-treated control groups, similar to our results [31, 52]. CsA has been reported to protect human conjunctival epithelial cells from cell death by preventing both extrinsic and intrinsic apoptosis pathways, consistent with the TUNEL staining results in the present study [53]. Together, our findings demonstrate that topical CsA, especially in SNEDDS preparations, decreases corneal epithelial cell death and increases cellular proliferation to restore damaged cells in BAC-induced DED.

Goblet cells lining the surface of the conjunctiva secrete MUC5AC, the gel-forming mucous element of the tear film [54]. We observed significantly fewer PAS^+^ goblet cells in murine DED, which were significantly increased by topical CsA treatment, consistent with a previous study [54]. Our fluorescein staining score results suggest that these goblet cells secreted sufficient mucins to stabilise the tear film. In DED patients with or without Sjögren syndrome, T cells infiltrate the conjunctiva [55, 56]. Proinflammatory cytokines released by activated T cells, such as interferon-γ, change the differentiation pattern of the mucosal epithelia, including conjunctival goblet cells [57, 58]. By suppressing T cell activation, CsA may prevent production and release of various cytokines and other mediators, including interferon-γ, restoring the integrity of goblet cells.

The current study has some limitations. Since we could not obtain the three cyclosporine vehicles, we were unable to compare and exclude completely vehicle effects. Therefore, in this study, we compared the physiochemical characteristics of three different cyclosporins. In addition, in the BAC induced dry eye murine model, some signs of dry eye disease, such as decreased tear production, low goblet cell density, and pro-inflammatory cytokine induction were observed, but have not yet been validated for CD4 + T cell or IL-2 introduction. Therefore, it was not clear whether SNEDDS-preparations are better at inhibiting T cell responses and IL-2 expression than C-CsA. This issue should be addressed in future studies.

The present study demonstrated that topical SNEDDS CsA eyedrops had improved pharmacochemical properties and *in vivo* clinical efficacy relative to C-CsA. Further, the SNEDDS CsA preparations protected the corneal epithelium and conjunctival goblet cells by suppressing inflammation more efficiently than C-CsA in this acute model and course of treatment. This information is relevant to clinical management of DED, as topical SNEDDS CsA preparations have enhanced physiochemical properties that decrease the induction period of CsA therapy.

## Supporting information

S1 Fig Gel and western blot images. Raw gel and blot images of Fig 8B.
(TIF)

S1 File The raw excel file of Ki-67 positive cells. Raw excel file of Fig 6A.
(XLSX)

S2 File The raw excel file of Ocular staining score. Raw excel file of Fig 3D.
(XLSX)

S3 File The raw excel file of PAS-positive cells. Raw excel file of Fig 7.
(XLSX)

S4 File The raw excel file of Tear volume measurements. Raw excel file of Fig 3E.
(XLSX)
