# Supplementary figures and images for: Cyclosporine A eyedrops with self-nanoemulsifying drug delivery systems have improved physicochemical properties and efficacy against dry eye disease in a murine dry eye model
Source: PLoS One. 2019 Nov 18;14(11):e0224805. doi: 10.1371/journal.pone.0224805 (PMC6860930; doi:10.1371/journal.pone.0224805)

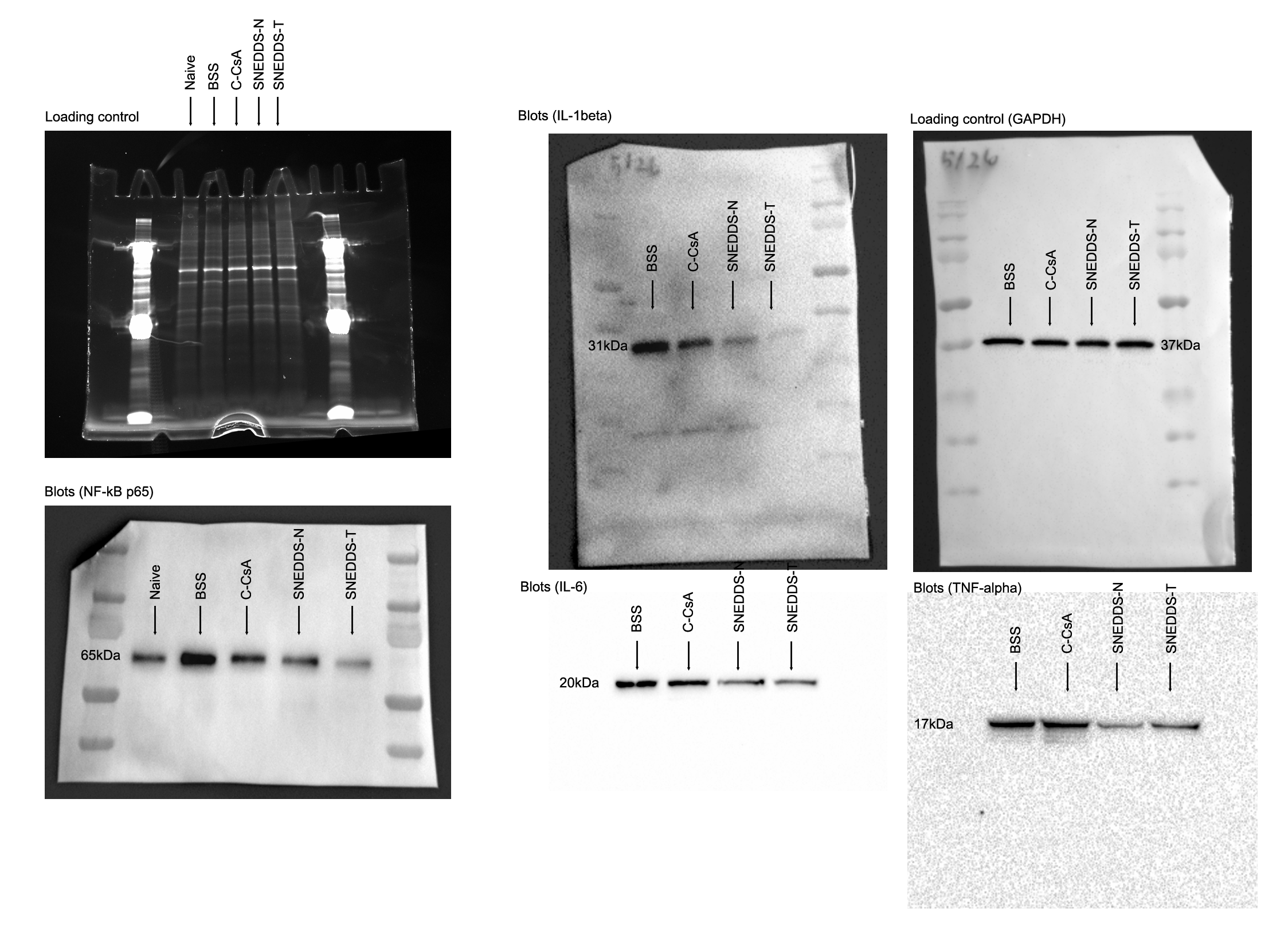

Supplement: S1 Fig — Raw gel and blot images of Fig 8B. (TIF) [file pone.0224805.s001.tif]
